# Can a brace be used to control the frequencies of a plate?

**DOI:** 10.1186/2193-1801-2-558

**Published:** 2013-10-24

**Authors:** Patrick Dumond, Natalie Baddour

**Affiliations:** Department of Mechanical Engineering, University of Ottawa, 161 Louis Pasteur, CBY A205, K1N 6N5 Ottawa, Canada

**Keywords:** Frequency matching, Assumed shape method, Brace-plate system, Musical instrument, Plate vibration, Orthotropic material, Tuning, Design-for-frequency, Spectrum control

## Abstract

Although many improvements in the manufacturing of guitars have been made recently, one aspect that has often been overlooked is that of the acoustical consistency of the final manufactured product. The aim of this paper is to create a better understanding of the effect of a brace on the frequencies of vibration of the brace-soundboard system. This paper seeks to shed light on why a luthier ‘tunes’ braces when a guitar soundboard is hand-manufactured. A simple analytical model of a rectangular brace and soundboard is derived from first principles using Kirchhoff plate theory in order to develop insight into the effect of the soundboard’s stiffness and brace thickness on the frequencies of the combined system. Natural frequencies and modeshapes of the combined system are calculated via the assumed shape method. Results show that by adjusting the thickness of the brace in order to compensate for the stiffness of the plate, one of the natural frequencies of the combined system can be adjusted to meet a desired value. However, simultaneously adjusting several natural frequencies cannot be done with a rectangular brace. Therefore modifications to the shape of the brace are explored.

## Introduction

The scientific study of guitars and other stringed musical instruments has been around for over a half century and many improvements to their production manufacturing have also been made (Richardson 1990; Chaigne 1999; French 2008a). In spite of this, manufactured instruments often do not sound as good as instruments built by hand by experienced luthiers. With precision tooling, production manufactured instruments can be built to strict dimensional tolerances and yet, acoustical consistency of the final product is still not ensured – meaning that two instruments emerging from the same production line will be dimensionally identical but acoustically different (French 2008b). There are two principal reasons for the lack of acoustical consistency. The first is that wood is a natural material, with natural variations so that soundboards that are dimensionally identical may be acoustically quite different. The other reason is that the tuning process used by the experienced luthiers to hand-build and tune instruments is based on years of experience and tradition but scientifically is not well understood. This tuning process is what luthiers use to make up for the natural variation in the acoustical properties of wood and since this tuning process is not well understood analytically, it is impossible to replicate autonomously. The goal of this paper is thus to develop a better analytical understanding of the tuning process of a guitar soundboard during its manufacture. The ultimate end goal is that this understanding can eventually be used to develop an automated tuning process to be used during guitar manufacture.

**Figure 1:**
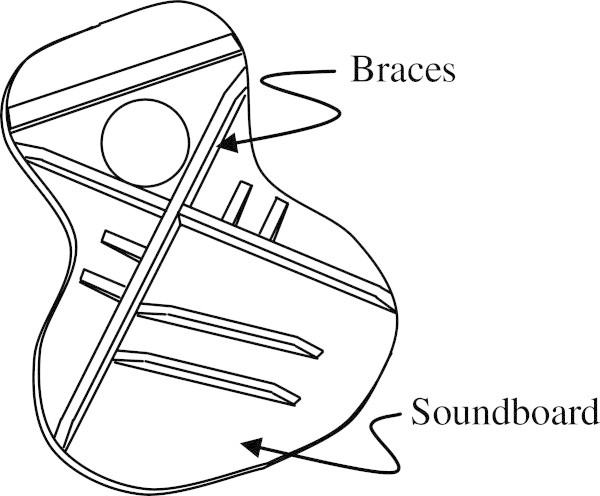
**Underside of a braced guitar soundboard.**

While all parts of a guitar contribute to the overall sound, there is general agreement that it is the soundboard, also known as the top plate, of the guitar that is most acoustically active and for which the highest inconsistency exists (Siminoff 2002a). The design of the soundboard serves two purposes, one structural and one musical. The first purpose is to structurally resist the immense tension of the strings and the second purpose is to produce the sound associated with the guitar. In order for the soundboard to be flexible enough to vibrate at the desired frequencies, it is quite thin. This makes the soundboard structurally unsuitable to resist the immense string tension. In order to compensate for this, braces are added to the underside of the soundboard as seen in Figure 1. While their function is primarily structural, they can also be used to adjust the frequencies of the soundboard-brace system. Soundboard frequency adjustments are known as tuning and this can be effectively done by varying the number and position of braces, as well as by changing their dimensions. For luthiers trying to follow a traditional bracing pattern, adjustment of the dimensions of these braces is generally the preferred method by which a guitar soundboard is tuned (Siminoff 2002b).

**Figure 2:**
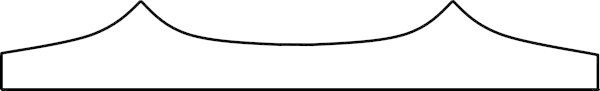
**Shape of a scalloped brace.**

The method most widely used by luthiers for optimising the brace and soundboard combination is known as tap tuning. Tap tuning involves listening to the change in frequency caused by the removal of brace material. The material is removed from the brace by hand and the tap-tuning process is an iterative process which involves tapping, listening and removing of brace material. By removing material from the brace, its stiffness is reduced as well as its mass; however the removal of wood has a larger impact on stiffness than on mass so the natural frequencies are reduced (Elejabarrieta et al. 2000). Since there is common consensus that the lower modes of vibration are of greater importance, generally only the lower natural frequencies are observed (Richardson 1990; Hutchins & Voskuil 1993; Natelson & Cumpiano 1994). This optimisation process often leads to what is known as scalloped braces, as shown in Figure 2. Much debate still exists as to the acoustic benefits of scalloped braces and the whole tuning process is still not well understood (Siminoff 2002b).

On the other hand, for production-manufactured instruments, current practice in the industry is to test the acoustic quality of a soundboard by measuring its stiffness across the grain during the manufacturing process (French 2008a). The deflection across the grain of the soundboard without braces is measured under a known load. Based on a certain set of deflection ranges, the soundboards are judged to be of higher or lower acoustical quality and are therefore used in different product lines. This test gives an idea as to the soundboard’s stiffness and thus the resulting frequency range of the final product. Based on the soundboard’s stiffness test, a brace from a collection of pre-dimensioned braces is chosen for that soundboard. A certain quality control is obtained since the manufacturer is aware of the range of soundboard stiffness for which their dimensioned braces produce decent instruments.

The fact that dimensional changes to the braces have a substantial impact on the frequency spectrum of the guitar is known but the extent of this effect is still unknown. However, research into the acoustics of musical instruments has begun to explain the fundamental interaction between various components of an instrument (Caldersmith 1995) and also the effect on the resulting sound field. Loosely generalized, research into guitars and other instruments has focused on their radiated sound fields or on their modal properties. Over the years, several experimental and computational techniques have been applied to the study of guitars, and in particular soundboards. One area of investigation has been the visualization of guitar box and soundboard resonances via holography and laser interferometry (Jansson 1971; Stetson 1981; Firth 1977; Richardson 2010; Jovicic 1977).

The radiation fields of the guitar have also been studied numerically and experimentally (Brooke & Richardson 1993;a; Brooke & Richardson 1991;b; Lai & Burgess 1990; Hill et al. 2004). Recent work has demonstrated that a relatively small number of measured parameters are required to predict the sounds radiated by a guitar (Richardson 2010; Hill et al. 2004; Richardson 2005). Interestingly, Brooke and Richardson conclude that there are no simple relationships between the modal properties of instruments and their estimated “quality” (Brooke & Richardson 1993a). At first glance this is surprising, although this is likely the scientific equivalent to the musical statement that just because an instrument is in tune does not ensure it is a “quality” instrument. On the other hand, most musicians will reject out of tune instruments as unplayable therefore one might infer that “good” modal properties are necessary but not sufficient to ensure a decent instrument. However, there does not seem to be a consensus on what constitutes “good” modal properties.

Elejabarrieta, Ezcurra, and Santamaría performed an extensive set of experimental and numerical studies on a guitar through its entire construction process by a master luthier. They experimentally tested the soundboard at each construction step as modifications were made by the luthier, in order to understand the effect of his modifications on the vibration properties of the soundboard (Elejabarrieta et al. 2000). This was followed by a finite element analysis of the same data (Elejabarrieta et al. 2001). As the guitar continued to be built by the same luthier, they continued with their work by analyzing the resonance box and its modes of vibrations, along with the effect of the sound-hole on the acoustic modes of the box (Elejabarrieta et al. 2002a). Their next step was an experimental and finite element analysis of the coupling of the vibration modes of the structural (soundboard and back-plate) and acoustic (the box as a Helmholtz resonator) modes once the box of the guitar had been assembled (Elejabarrieta et al. 2002b). Finally, they investigated the fluid-air interaction of the guitar box in which the interior gas was changed both experimentally and numerically (Ezcurra et al. 2005). This set of work contributed greatly to the understanding of how each component of the guitar contributes to the final frequencies of vibration but did not offer any suggestions as to how an instrument designer might modify a given component to achieve a specific acoustical objective. Boullosa experimentally measured the radiation efficiency and frequency content of the vibrations of a classical guitar (Boullosa 2002; Boullosa et al. 1999) but offers little in the way of insight as to which components of the guitar or their modifications contribute to either. Torres and Boullosa also studied the effect of the bridge on the vibrations of the soundboard both with finite elements and with laser vibrometry (Torres & Boullosa 2009). Chaigne and various collaborators focused their research on the time-domain modelling of the guitar (Chaigne 1999; Bécache et al. 2005; Derveaux et al. 2003) with the intent to better understand the vibroacoustical behavior via physical and numerical modeling. Their intent was for this to be used as a tool for the estimation of quantities that are hard to measure experimentally as for example the estimation of the relative structural losses and radiation losses in the sounds generated by the guitar.

In other (non-musical) disciplines, stiffened plates have been previously studied by various methods (Fox & Sigillito 1980; Barrette et al. 2000; Peng et al. 2006; Hong et al. 2006). However these studies have focused on the structural properties of such systems rather than the acoustical properties of the interaction between the beam and plate elements.

Despite the work that has been done to understand the mechanics of the instruments, little has been done to improve the acoustical consistency of manufactured guitars in large part because the tuning process that is used for hand-built instruments is not well understood and thus cannot easily be replicated. The goal of this paper is to begin to develop a better understanding of this tuning process via a simple analytical model to represent the vibrations of an instrument soundboard and a supporting/tuning brace. A simple model is sufficient to answer the question at hand, therefore a simple model that can yield the most physical insight is chosen for the analysis. The question is: can we change the dimensions of the brace to make up for changes in stiffness of the soundboard so that the combined brace-soundboard system has desired frequencies of vibration? This is the essence of what luthiers do when they hand-tune an instrument during its construction; they make small changes to the structural properties in order to produce desired changes in acoustical properties. An idea similar to this has been considered analytically for a xylophone (Orduna-Bustamante 1991), where the effect of an undercut on the bar on its tuning was modelled and analyzed. However, to the best of the authors’ knowledge, this type of analysis has not been considered for a guitar.

Current research shows that it is the first few modes of the coupled system that are necessary in order to tune the soundboard during manufacturing (Hutchins & Voskuil 1993). In this work, the assumed shape method is used to analyze the continuous system, with the modes of the soundboard without the brace used as the solution building blocks. The assumed shape method is a global element method detailed in (Meirovitch 1996a). It has been shown to be particularly usefully in the modal analysis of stiffened and orthotropic plates (Xu et al. 2010; McIntyre & Woodhouse 1988). The assumed shape method has specifically been chosen as the analysis tool because it can solve the mass and stiffness matrices analytically; thereby each matrix entry is a function of *all* physical parameters. This helps give vibration insight about the model’s natural frequencies and corresponding modes (Bisplinghoff et al. 1996).

A brief outline of the paper is as follows. Section 2 presents the analytical model, specifically chosen based on the pertinent background information provided in Section 1. Section 3 demonstrates the results of the research and Section 4 is a discussion of the results. The effects of scalloped braces are dealt with in Section 5. Finally, conclusions are given in Section 6.

## Analytical model of the plate-brace system

### A. Modeling assumptions

**Figure 3:**
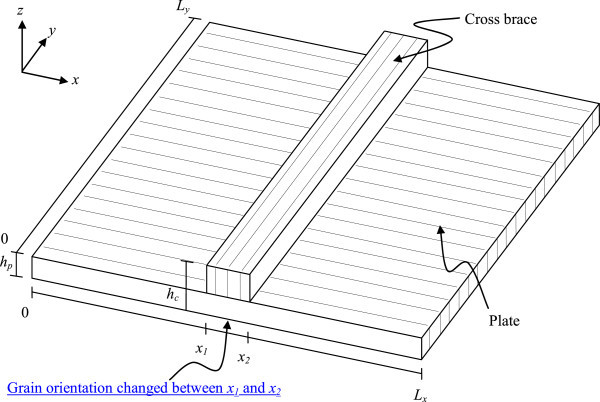
**Orthotropic rectangular plate fitted with brace across its width.**

To analytically investigate the brace/soundboard interaction, we analyze the natural frequencies and modeshapes of a rectangular plate model with an attached cross-brace, as shown in Figure 3.

The soundboard is modeled as a thin rectangular Kirchhoff plate and the brace is modeled as a thicker section of the same plate. A simple rectangular geometry is assumed in order to enable the closed-form solution of a simple plate (without the brace) to be used as the trial functions for the assumed shape method. Further, since the solution of a rectangular plate is known in closed form, this will enable a direct comparison and enable the understanding of the effect of the brace on the vibration properties of the plate.

The Kirchhoff plate theory assumes small transverse deflections and neglects transverse normal and shear stresses, as well as rotary inertia. Although this is an accurate assumption for the plate, due to the brace’s thickness-to-width aspect ratio, it may imply a certain error in that region of the soundboard. Also, because of the method in which the brace thickness is added to that of the plate in the kinetic and strain energy expressions, it was necessary to change the direction of the grain of the plate, in this region only, to match that of the brace. This is reasonable since the plate is thin and the properties of the brace dominate in this region. The plate is also assumed to be simply supported all around, although in reality it is somewhere between simply supported and clamped (Meirovitch 1996b). It has been assumed that the system is conservative in nature, which allows damping to be neglected. Although there is a certain amount of damping found in wood, its effects on the lower natural frequencies is thought to be minimal and has been neglected. This is justified because the tuning process (adding and adjusting the dimensions of the braces) has a greater effect on the lower frequencies than on the higher frequencies (Hutchins & Voskuil 1993).

The orthotropic properties of wood are modeled, therefore its longitudinal and radial properties are of interest, labelled *L* and *R* respectively. The only material properties that need to be considered independently in these directions are Young’s modulus, *E* and Poisson’s ratio, *ν*. For an orthotropic plate the stress–strain relationships are given by (Riley et al. 2006)1

where the *S* are stiffness components are2

The subscripts represent the direction of the plane in which the material properties act. Therefore, *E*_*x*_ is the Young’s modulus along the *x*-axis, *E*_*y*_ along the *y*-axis and *ν*_*xy*_ and*v*_*yx*_ the major Poisson’s ratios along the *x*-axis and *y*-axis respectively. We begin with the expressions for strain and kinetic energies for an orthotropic plate and then consider modifications to these when the brace is added to the plate.

### B. Strain energy for an orthotropic plate

Using the stress–strain relationships of Eq. (1), the strain energy for an orthotropic plate is given by (Timoshenko & Kreiger 1964).3

where *L*_*x*_and *L*_*y*_are the dimensions of the plate in the *x*and *y*directions, *w* = *w*(*x*, *y*)is the transverse displacement and the subscripts on *w* refer to partial derivatives in the given direction. The plate’s stiffnesses *D*are given by4

where *h*is the thickness of the plate.

### C. Kinetic energy for an orthotropic plate

The orthotropic properties of the plate only affect its stiffness and not its density, so its kinetic energy is the same as for an isotropic plate5

where the dot represents the time derivative, *ρ* is the mass per unit area of the plate such that *ρ* = *μ* ⋅ *h*, *μ* is the material density and *h* is the plate’s thickness.

### D. Strain energy for a plate modified with an attached brace

To account for the modification of the plate by adding a brace to it, as seen in Figure 3, the strain and kinetic energies are modified to account for additional thickness between *x*_1_ and *x*_2_.

From the expression for strain energy, Eq. (3), the only term affected by the change in thickness between *x*_1_ and *x*_2_ is the stiffness *D*. Therefore, the integral of Eq. (3) is split into three separate parts so that the strain energy becomes6

The stiffnesses *D* are now section-specific because of the change in thickness *h* from *x*_1_ to *x*_2_:7

and8

where the subscripts *p* and *c* denote the plate alone and combined plate-and-brace system.

### E. Kinetic energy for a plate modified with an attached brace

Similar to the method used to modify the strain energy term, the kinetic energy can also be written to take into account the change in thickness from *x*_1_ to *x*_2_:9

where the density per unit area *ρ* is now calculated as:10

### F. The assumed shape method

The assumed shape method is chosen because it allows us to use the flat-plate modeshapes as the fundamental building blocks of the solution, thereby permitting observation of how the addition of the brace affects those fundamental modeshapes. This method also permits greater flexibility in analyzing the effects of changes in brace dimensions since it enables the creation of an analytical solution from which numerical solutions can be quickly obtained for various thicknesses of the brace. The equations of motion are derived using a computer algebra system (Maple). This yields mass and stiffness matrices where each matrix entry is a function of *all* physical parameters (dimensions, density, stiffnesses, etc.). The effect of any parameter on the system’s eigenvalues can then easily be examined without having to re-establish the entire system model. Using the assumed shape method, it has also been found that only two additional odd or even trial functions more than the one of interest are required for convergence (depending on whether it is itself odd or even). The finite element method was also considered. While the FE method offers significant advantages over the other approximate methods, namely its ability to model complex systems and boundaries and a high numerical accuracy, its disadvantage for the purpose of this work is its inability to make use of the known mode shapes of the system without the brace. Furthermore, the FE method also requires a large number of degrees of freedom in order for the solution to converge to accurate results. Contrary to the nature of the global functions approach, the finite element method uses local functions which extend over small subdomains of the system (Meirovitch 1996b), thus comparison of the global behaviour to the exact solution of a simple plate problem cannot be directly incorporated into this solution approach.

The first step of the assumed shape method is to approximate the transverse displacement *w*(*x*, *y*, *t*) as (Meirovitch 2001)11

The  are the chosen discrete spatial trial functions and  are the generalized (time-dependent) coordinates. Also, *m*_*x*_ and *n*_*x*_ represent the mode number and trial function number in the *x* direction respectively and *m*_*y*_ and *n*_*y*_represent the same in the *y* direction. Next, the trial functions are chosen so as to satisfy the geometric boundary conditions and be complete in order to ensure convergence of the solution (Meirovitch 2001). No other considerations of the boundary conditions need to be taken into account. A simply supported plate implies boundary conditions such that the transverse displacement *w* of the perimeter of the plate is zero (Meirovitch 1996b).

Here, the modeshapes of the simply supported rectangular plate (without the brace) are known and these will be used as the trial functions in Eq. (11), so that12

Applying the trial functions of Eq. (12) to Eq. (11), gives a discrete series13

which is then used in the strain and kinetic energy equations of the modified plate.

Once the strain and kinetic energies have been assembled, Lagrange’s equations are used to find the equations of motion which can then be written in matrix form as14

where *M* is the mass matrix and *K* is the stiffness matrix given. Additionally,  is the generalized coordinate vector15

Letting the generalized coordinate system have a harmonic solution as in (Meirovitch 1996b), then16

Here, *ω* is the system’s natural frequency, *ϕ* the phase shift and  is a magnitude vector of dimension(*m*_*x*_ ⋅ *m*_*y*_) × 1. Then replacing the assumed harmonic solution into the equation of motion, Eq. (14) an eigenvalue problem is obtained, from which the natural frequencies and modeshapes are found.

## Results

The purpose of this analysis is to verify if it is possible to alter the dimensions of the brace so as to obtain a desired set of natural frequencies from the coupled system, knowing their respective properties before assembly. Both symbolic and numerical computational tools are used.

### A. Material properties

The material used throughout the analysis is that of Sitka spruce, the most commonly used wood for stringed musical instrument soundboards. Material properties for Sitka spruce are obtained from the U.S. Department of Agriculture, (Forest Products Laboratory (US) 1999). Since properties between specimens of wood have a high degree of variability, the properties obtained from the Forest Products Laboratory are an average of specimen samplings. The naturally occurring properties of wood act as an orthotropic material. Material properties of Sitka spruce are seen in Table 1. The subscripts ‘*R*’ and ‘*L*’ refer to the radial and longitudinal property directions of wood respectively.

**Table 1:** **Material properties for Sitka spruce as an orthotropic material (Forest Products Laboratory (US)** 1999 **)**

Material Properties	Values
Density – *μ* (kg/m^3^)	403.2
Young’s modulus – *E*_*R*_ (MPa)	850
Young’s modulus – *E*_*L*_ (MPa)	*E*_*R*_ */*0.078
Shear modulus – *G*_*LR*_ (MPa)	*E*_*L*_ *×* 0.064
Poisson’s ratio – *ν*_*LR*_	0.372
Poisson’s ratio – *ν*_*RL*_	*ν*_*LR*_ *× E*_*R*_ */E*_*L*_

### B. Plate and brace dimensions

**Figure 4:**
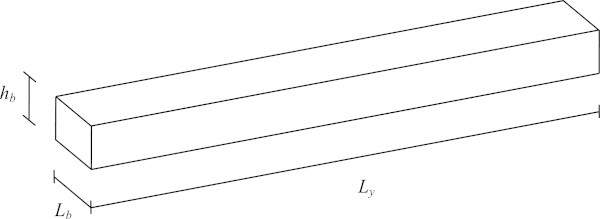
**Brace showing pertinent dimensions.**

A control test specimen, having the same dimensions for every analysis, is used. The dimensions of the plate and brace are based on typical dimensions of a section of instrument soundboard for which a single brace is used for structural reinforcement. This turns out to be about a quarter of a typical guitar soundboard. The brace dimensions are defined as in Figure 4.

The plate dimensions are defined as in Figure 3. Other important reference points are also indicated. All the dimensions used in this study for the brace and the plate as well as other pertinent reference points are listed in Table 2. To avoid confusion, subscript ‘*p*’ stands for plate, ‘*b*’ for brace and ‘*c*’ for combined plate and brace.

**Table 2:** **Test specimen dimensions**

Dimensions	Values
Length – *L*_*x*_ (m)	0.24
Length – *L*_*y*_ (m)	0.18
Length – *L*_*b*_ (m)	0.012
Reference – *x*_*1*_ (m)	*L*_*x*_ */*2 *– L*_*b*_ */*2
Reference – *x*_*2*_ (m)	*x*_*1*_ *+ L*_*b*_
Thickness – *h*_*p*_ (m)	0.003
Thickness – *h*_*b*_ (m)	0.012
Thickness – *h*_*c*_ (m)	*h*_*p*_ *+ h*_*b*_

### C. Frequencies and modeshapes

The assumed shape method used 10 × 10 trial functions. Table 3 gives these results of this modelling approach. Additionally, results of the natural frequencies obtained via the assumed shape method for the first ten modes were compared to those obtained using the finite element method with over 21000 nodes. This served to verify and validate the results presented here.

**Table 3:** **Results for the assumed shape with 10x10 trial functions (orthotropic)**

***m***_***x***_	***m***_***y***_	Assumed shape method	
		**Natural Frequency (Hz)**	**Modeshape**
1	1	590	
2	1	703	
2	2	930	
1	2	1015	
2	3	1185	
1	3	1248	
3	1	1273	
2	4	1551	
1	4	1598	
2	5	2051	

The dip in the center of the *x*-axis for the assumed shape method modeshapes is the location of the brace, which is visible on the modeshapes. The brace stiffens this area and limits the amount of displacement that can occur. Since the Kirchhoff model is used, shear and rotary inertia are neglected, which may induce some inaccuracies near the brace as the aspect ratio is larger there.

### D. Effect of the brace on the natural frequencies of the combined system

Systems having components combined using a rigid link have the sum of the stiffness and of the mass components of each subsystem, leading to natural frequencies which generally fall somewhere between the two separate systems’ original natural frequencies. To verify this claim on the continuous system, the original orthotropic system’s natural frequencies are compared to those of the combined orthotropic system in Table 4. The exact values for the natural frequencies of the brace seen in Figure 4 using classical beam theory are given in (Hartog 2008). The exact values for the natural frequencies of the simply supported orthotropic plate of Figure 3 are calculated via the assumed shape method using the exact modeshapes obtained from (Meirovitch 1996b).

**Table 4:** **Comparison of the orthotropic brace, plate and combined system natural frequencies**

***m***_***x***_	***m***_***y***_	Brace natural frequencies	Plate natural frequencies	Combined system natural frequencies	% Increase by adding
		(Hz)	(Hz)	(Hz)	the brace
1	1	873	166	590	256%
2	1	873	530	703	33%
2	2	3491	662	930	40%
1	2	3491	330	1015	207%
2	3	7855	923	1185	28%
1	3	7855	629	1248	98%
3	1	873	1146	1273	11%
2	4	13965	1321	1551	17%
1	4	13965	1054	1598	52%
2	5	21820	1853	2051	11%
1	5	21820	1604	2090	30%

These results show intuitive trends which help verify the model and help in the understanding of the effect that adding a brace has on the coupled system. A detailed discussion about the relationship between these results is given in Section 4.

### E. Tuning

In order to verify the feasibility of tuning braces to a plate having a predetermined cross-grain stiffness, it is necessary to look at effects of a change in both the Young’s modulus in the radial direction *E*_*R*_ and of the brace thickness *h*_*b*_, on the modified orthotropic plate of Figure 3. Although the lowest five natural frequencies carry importance, only two will be observed during the variation in structural properties. This is because frequencies that have a mode of vibration which contains a node at the location of the brace are not as affected by the brace as those which have a mode which passes through it. Therefore the two frequencies observed during this analysis are the first and fourth natural frequencies of the orthotropic plate-brace system. The second and third modeshapes have a node at the location of the brace and are not as affected by the brace, contrary to the first and fourth modeshapes which don’t. This can be observed in Table 4, where the first and fourth modes use only one trial function along the *x*-axis such that *ω*_1_:*m*_*x*_ = 1, *m*_*y*_ = 1 and *ω*_4_:*m*_*x*_ = 1, *m*_*y*_ = 2.

**Figure 5:**
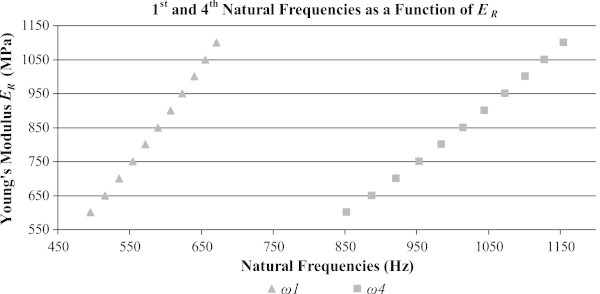
**The 1st and 4th natural frequencies of the combined system when varying** ***E***_***R***_ **(** ***h***_***b***_ **= 0.012 m).**

Since it seems that the cross-grain stiffness of a soundboard has a large impact on its acoustical properties and since this stiffness is related to the soundboard’s radial Young’s modulus, the radial Young’s modulus or *E*_*R*_ is varied to see its effect on the systems natural frequencies. The brace is kept to a constant thickness of *h*_*b*_ =0.012m. The results of this are shown in Figure 5.

**Figure 6:**
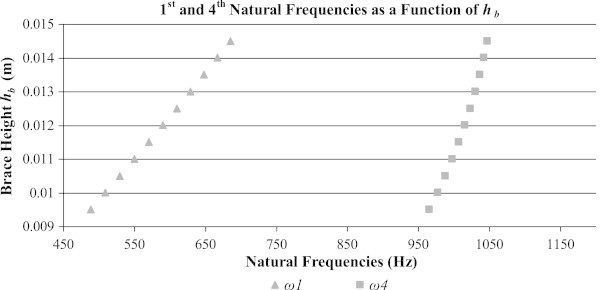
**The 1st and 4th natural frequencies of the combined system when varying** ***h***_***b***_ **(** ***ER = 850 MPa).***

It is clear from Figure 5 that as *E*_*R*_ increases, so do the 1st and 4th natural frequencies. A similar analysis is again performed, but this time *E*_*R*_ is held constant at 850MPa and the thickness of the brace or *h*_*b*_ is varied. These results are shown in Figure 6.

In the same way as the previous case, it can be seen from Figure 6 that when *h*_*b*_ increases so do the 1st and 4th natural frequencies of the combined system.

Based on these results and in order to verify if it is possible to get consistency out of the natural frequencies, an analysis was performed in which an increase in the plate’s radial stiffness was compensated by reducing the thickness of the brace, shown in Table 5. The plate’s radial stiffness and the thickness of the brace were varied so as to keep the 1st natural frequency relatively constant while examining the effect this would have on the 4th natural frequency.

**Table 5:** **The system is compensated so that the 1st natural frequency is held constant**

Young’s modulus	Brace thickness	1st natural frequency	% change of ***ω***_***1***_from	4th natural frequency	% Change of ***ω***_***4***_from
***E***_***R***_(MPa)	***h***_***b***_(m)	***ω***_***1***_(Hz)	***E***_***R***_ = 850	***ω***_***4***_(Hz)	***E***_***R***_ = 850
600	0.0150	590	0.0%	884	13%
650	0.0142	590	0.0%	913	10%
700	0.0136	592	0.3%	941	7%
750	0.0130	591	0.2%	967	5%
800	0.0125	592	0.3%	992	2%
850	0.0120	590	0%	1015	0%
900	0.0116	591	0.2%	1038	2%
950	0.0112	590	0.0%	1059	4%
1000	0.0109	592	0.3%	1080	6%
1050	0.0105	589	0.2%	1098	8%
1100	0.0102	589	0.2%	1117	10%

It is apparent from Table 5 that although the first natural frequency has been held more or less constant, this also resulted in significant variation in the fourth natural frequency. This led to a further analysis in which the radial stiffness and brace thickness were varied so that the fourth natural frequency was held constant, as shown in Table 6.

**Table 6:** **The system is compensated so that the 4th natural frequency is held constant**

Young’s modulus	Brace thickness	1st natural frequency	% change of ***ω***_***1***_from	4th natural frequency	% change of ***ω***_***4***_from
***E***_***R***_(MPa)	***h***_***b***_(m)	***ω***_***1***_(Hz)	***E***_***R***_ = 850	***ω***_***4***_(Hz)	***E***_***R***_ = 850
600	0.0909	934	58%	1015	0.0%
650	0.0683	927	57%	1015	0.0%
700	0.0387	907	54%	1015	0.0%
750	0.0194	781	32%	1015	0.0%
800	0.0144	661	12%	1015	0.0%
850	0.0120	590	0%	1015	0%
900	0.0104	541	8%	1015	0.0%
950	0.0093	508	14%	1015	0.0%
1000	0.0084	482	18%	1014	0.1%
1050	0.0077	462	22%	1014	0.1%
1100	0.0071	446	24%	1014	0.1%

Once again, forcing the fourth natural frequency to be more or less constant causes the first natural frequency to vary considerably from its value at *E*_r_ = 850MPa. These results are further discussed in the next section.

## Discussion

Based on the results presented in Section 3, the analysis has demonstrated clear trends in the behaviour of a soundboard having a brace across its width. Specific points are discussed herein.

### A. Material properties

The first thing to note during this analysis is the use of the statistical average values of spruce’s material properties. It is obvious that these material properties vary on a specimen by specimen basis. However the assumption was made that there is a relationship between the radial stiffness *E*_*R*_ and the other properties. While this is definitely alluded to by the (Forest Products Laboratory (US) 1999), it is unclear how much variation is actually present in these relationships. Based on years of luthier experience in using the cross-grain stiffness as a measure of soundboard quality, it would appear that the relationship between this stiffness and other properties is more consistent than the properties themselves. It would, however, be quite interesting to further investigate this phenomenon, as this is has been found to be a great way of modeling the material properties of wood.

Clear, quartersawn, musical instrument spruce has been shown to have remarkably consistent microscopic properties in spring and summer growth. Furthermore, spruce displays an abrupt transition period which means that micro-scale properties are generally in line with macro-scale mechanical properties (Kahle & Woodhouse 1994). Therefore, by avoiding visual imperfections as is currently being done in industry, it is reasonable to assume that bulk material properties taken on a specimen-by-specimen basis is an adequate measure of overall system performance. However, it would be interesting to investigate how localized changes in material properties would affect system performance, especially at the location of the brace. Modifications to the method, by increasing the number of zones of interest in the kinetic and strain energy equations for example, could take into account micro-scale variations in the material specimen. On the other hand, more rigorous testing of material properties would be necessary to populate the input information required for the analysis. On the macro-scale, CNC machinery could be reprogrammed to shape braces as required by the material property measurements of the plate.

To increase the accuracy of the model by including frequency-dependent damping properties, an analytical approach such as that described in (McIntyre & Woodhouse 1988) could be used to modify the mathematical model in order to take damping into account.

### B. Dimensions

The dimensions used on the test specimen consisting of the simply supported rectangular plate and brace across the width, are based on typical dimensions of those used on guitar soundboards. The plate itself having only one brace is typical of the area on a soundboard around the lower bout where is positioned one of the legs of the typical x-brace pattern. This leads to a model which produces a set of the lowest frequencies within the acoustical range sought by a typical musical instrument (e.g. A0-C8 or 27.5 − 4186.01*Hz*).

### C. Effect of the brace on plate modes

When results are compared between the plate alone and the plate with the brace as seen in Table 4, it is clear that the addition of the brace affects modes for which the location of the brace is not a node, such as *m*_*x*_ = 1, more than a mode having a node at the location of the brace, such as *m*_*x*_ = 2. This is also clear from the percentage increase in the natural frequencies for the *m*_*x*_ = 1 mode, which is much higher than for the *m*_*x*_ = 2 modes. It is also impossible to avoid a slight increase in the natural frequencies for the *m*_*x*_ = 2 modes or all other even modes because their nodes are along a line and the brace does in fact have a finite width. This finite brace width causes local stiffening to occur around it, affecting the curvature of the even modes and thereby also increasing their frequencies.

This table also demonstrates that for modes directly affected by the brace, the natural frequencies in fact fall somewhere between those of the brace and plate alone. These results are those which are expected from basic vibration theory. For the modes not directly affected by the brace, a simple increase in the original plate’s natural frequency is observed due to the forced changes in the mode’s curvature.

### D. Tuning

The effects of variations in the cross-grain stiffness of the plate, measured as *E*_*R*_, are observed in Figure 5. It is clear that when the stiffness across the grain is reduced so too are the natural frequencies, as expected. Similarly, the effects of the brace’s thickness, measured as *h*_*b*_, are considered in Figure 6. As the brace’s thickness increases, so does its natural frequencies since its stiffness increases at a larger rate than its mass.

Luthiers use this phenomenon in order to adjust the braces to a given soundboard, changing the thickness of the brace in certain sections to compensate for changes in the stiffness of the soundboard. This adjustment process is tested analytically whereby the brace is adjusted inversely to the plate’s cross-grain stiffness in order to hold the first natural frequency of the combined system constant. These results can be seen in Table 5. While the first natural frequency variation falls well below the 1% human hearing threshold for sound variation (Chaigne 1999), the variation in the fourth natural frequency lies above it.

A second attempt was made to tune the fourth natural frequency, modifying the properties of the brace so that the fourth natural frequency remained constant despite variations in the stiffness of the soundboard. As can be seen in Table 6, a wider adjustment span is required for the brace in order to achieve consistency in the fourth natural frequency. This time, the frequency variation of the fourth natural frequency lies well below the 1% threshold. However, the variation in the first natural frequency is wider than the first attempt.

These results indicate that it is possible to produce an acoustically consistent set of brace-plate assemblies that have at least one desired natural frequency. Conversely, it does also indicate that a rectangular brace is not suitable for adjusting multiple frequencies.

After obtaining these results, it has become clear that adjustments to the shape of the brace itself are required. This has led back to the debate on whether or not scalloped braces have an acoustical role in producing more consistent instruments. This being an interesting topic on its own, further investigation of the scalloped brace is found in Section 5.

### E. Sources of error

Evidently, improved accuracy in the calculation of the natural frequencies of the modified plate could be obtained by simply incorporating shear deformation and rotary inertia into the plate model. Nevertheless, other assumptions were also made which have an impact on the preciseness of the calculated values.

The first assumption was that the mass of air which would normally surround the soundboard of a musical instrument has been neglected. Including the mass of air surrounding the soundboard would in fact decrease the natural frequencies because the mass of air acts to increase the total inertia of the soundboard (Leissa 1993).

To simplify the model, the assumption was also made that the soundboard is simply supported when in fact it is probably somewhere between simply supported and clamped (Meirovitch 1996b). Since clamped edges prevent rotation at the edge, local stiffening occurs. This leads to an increase in the natural frequencies.

Damping was also neglected during the analysis, which allowed for a much simpler model. Although this assumption is justified due to the fact that a musical instrument is designed to sustain rather than to absorb vibration, it is the damping or decay time of specific partial frequencies, because of wood’s distinct properties, which help give a wooden instrument its tone (Chaigne 1999). Therefore, to improve the acoustical preciseness of the model, damping would need to be included in the analysis.

Finally, in order to create the orthotropic system, the direction of the grain at the location of the brace was changed for the plate. This would in fact slightly modify the stiffness properties of the system in this location, thereby influencing the natural frequencies.

## Analysis of a scalloped brace

Based on the results of Section 3, it is clear that a rectangular brace can be modified to control one of the lower frequencies, but alone is unable to control multiple frequencies at once. Based on these previous results, insight was obtained as to what needs to be done in order to tune at least two of the lower modes, therefore a preliminary analysis was performed based on the hypothesis that modifying the shape of the brace itself has the ability to control more than one of the system’s natural frequency.

**Figure 7:**
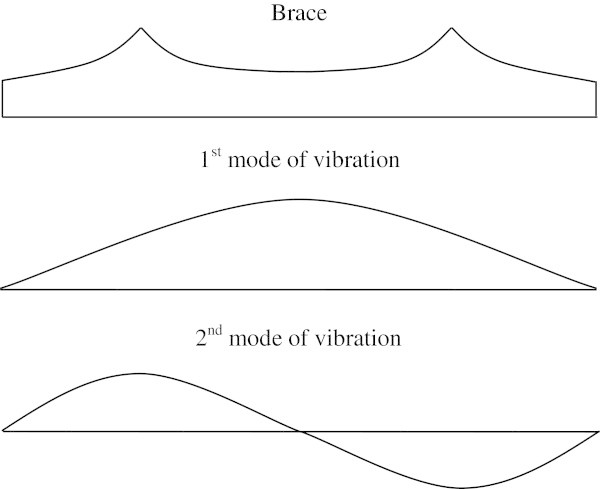
**Scalloped brace with the modes of vibration it affects.**

As previously mentioned, during the manual tuning process a brace will often end up having a scalloped shape. While some believe this is the result of the tuning process, there has been some speculation that this enables a luthier to control two modes at once (Siminoff 2002b). This speculation is based on the fact that because of a scalloped brace’s peculiar shape, individual modifications of the two lowest modes running along its longitudinal direction, as seen in Figure 7, are possible.

### A. Modeling of the scalloped brace

For comparative purposes, the same model used for the rectangular brace including the orthotropic material properties described will be used in this section. Only preliminary modifications to the shape of the brace itself are explored herein. In order to model the scalloped brace, a second order piece-wise polynomial function was chosen to model the thickness of the brace. This polynomial function puts the peaks of the scallops at ¼ and ¾ of the way down the brace. The function is given by17

where *h*_*bo*_ is the height of the brace at its ends and center. This *h*_*b*_ is then substituted into the kinetic and strain energy equations as used for the modified plate model in the assumed shape method.

### B. Results

Since the equations of kinetic and strain energy now include a polynomial instead of a constant during the solution process, the computational power necessary for such a symbolic solution increases immensely. The results obtained for the orthotropic modified plate using a scalloped brace can be seen in Table 7. The solution uses 5 × 5 trial functions in order to solve the lowest five natural frequencies. In order to compare results with the results of the previous section, the rectangular brace is given a thickness of *h*_*b*_ =0.012m, as before. The original thickness of the scalloped brace is also marked as *h*_*bo*_ =0.012m.

**Table 7:** **Comparison of brace geometry on a simply supported orthotropic modified plate**

***m***_***x***_	***m***_***y***_	System natural frequencies with rectangular brace	System natural frequencies with scalloped brace	% change in frequencies
		(Hz)	(Hz)	
1	1	592	621	4.9%
2	1	711	743	4.5%
2	2	950	1003	5.6%
1	2	1063	1053	−0.9%
2	3	1211	1279	5.6%

### C. Discussion

Although the natural frequencies have a clear increase in value throughout for the scalloped brace compared to the rectangular brace, there is a marked difference for the 1 × 2 mode, where there is in fact a reduction in the natural frequencies of the system. The original thickness of the scalloped brace is equal to that of the rectangular brace, which adds additional material to the system, and thus increases the natural frequencies. Since the peaks of the scalloped brace occur at the maximum displacement locations of the 4th mode of vibration in the direction of the brace (1 × 2 mode), it increases the inertia at these locations by increasing the mass locally. This is the reason for the reduction in the natural frequency observed for the 4th modeshape. These peaks also minimize the effect of extra mass on the other modes of vibration, because their maximum displacement is found to be either at the center of the brace where the brace’s thickness goes unchanged or the brace is in fact at a location of one of their nodes.

These discoveries lead to the theory that what a luthier is in fact doing when scalloping a brace, is adjusting two or more modes at once, or at least controlling which modes are affected the most by the bracing since not all modes are equally affected. It is evident that the exact shape chosen based on the polynomial of Eq. (17) may not be the optimal solution. Further investigation into the scallop shape itself is necessary to further grasp the magnitude of its effect on the frequency spectrum of the soundboard. Research on understanding the effects of a scalloped shape brace on the natural frequencies of a brace-plate system is ongoing, but shows great promise in a field that deserves to be explored. Only preliminary results have been discussed herein.

## Conclusions

In this paper, the effect on natural frequencies of adding a brace to a soundboard was modeled and analyzed in order to better understand how luthiers tune a musical instrument. The assumed shape method was used in the analysis and the insight gained by using this approach was tremendous.

First, the analysis has shown that the acoustic properties of a soundboard can be modified by adjusting the thickness of a brace. In fact, it has shown that specific natural frequencies can be controlled. However, the rectangular brace used for most of the analysis has been found to have the ability to control only one frequency at a time.

Scalloped braces have been shown to be a solution for which multiple natural frequencies of a soundboard can be adjusted. Further investigation on this subject should be explored. However, it does indeed help to clarify the purpose of using scalloped braces, as has been done for hundreds of years, and also gives hope that it is possible for a wooden musical instrument manufacturing process to include acoustical consistency.

## Nomenclature

A: Magnitude vector

*D*: Stiffness function

*E*: Young’s modulus

*G*: Shear modulus

*h*: Thickness

*k*: Stiffness

K: Stiffness matrix

*K*_*s*_: Shear correction factor

*m*: Mass

M: Mass matrix

*L*: Longitudinal axis parallel to the wood grain

*L*_*x*_: Length along the x axis

*L*_*y*_: Length along the y axis

*m*: Mode number

*n*: Trial function number

*q*: Generalized coordinates

*Q*: Generalized non-conservative forces

*R*: Radial axis normal to the growth rings and perpendicular to the wood grain

*S*: Stiffness components

*t*: Time

*T*: Kinetic energy of the system

*T*: Tangential axis tangent to the growth rings and perpendicular to the wood grain

*u*: Displacement along the x axis

*U*: Strain energy of the system

*v*: Displacement along the y axis

*V*: Potential energy of the system

*w*: Displacement along the z axis

*w*_*o*_: Displacement along the z axis of the plate’s neutral plane

*W*: Work

*x*: Axis direction and position

*y*: Axis direction and position

*z*: Axis direction and position

*γ*: Shear strain

*ϵ*: Normal strain

*μ*: Density

*ν*: Poisson’s ratio

*ρ*: Mass per length or mass per area

*σ*: Normal stress

*τ*: Shear stress

*φ*: Discrete spatial trial functions

*ω*: Natural frequency
